# Type 2 diabetes mellitus associated microRNAs in tuberculosis susceptibility: a systematic review and bioinformatic analysis

**DOI:** 10.3389/fendo.2026.1760933

**Published:** 2026-02-23

**Authors:** Rodolfo L. Chávez-Domínguez, Mercedes Viettri, Martha Torres, Itzel A. Corona-Galvan, Emilio Hernández-Diego, Mauricio Castañón-Arreola, Claudia Carranza

**Affiliations:** 1 Laboratorio de Inmunobiología de la Tuberculosis, Instituto Nacional de Enfermedades, Respiratorias Ismael Cosío Villegas, Ciudad de Mexico, Mexico; 2 Posgrado en Ciencias Genómicas, Universidad Autónoma de la Ciudad de México, Ciudad de México, Mexico

**Keywords:** bioinformatic analysis, biomarkers, microRNAs, systematic review, tuberculosis, type 2 diabetes mellitus

## Abstract

**Introduction:**

The coexistence of tuberculosis (TB) and type 2 diabetes mellitus (T2DM) represents a growing global health challenge, particularly in low- and middle-income countries where TB remains endemic and T2DM prevalence is rising. Patients with T2DM exhibit a threefold higher risk of developing active TB and frequently present with more severe disease, including increased bacillary burden, delayed culture conversion, and higher relapse rates. These outcomes reflect the complex immunometabolic interactions between the two conditions. MicroRNAs (miRNAs), small non-coding regulators of post-transcriptional gene expression, emerge as potential biomarkers capable of integrating immune and metabolic processes.

**Methods:**

we conducted a systematic review of studies published between 2011 and 2025 in PubMed and Google Scholar, following PRISMA 2020 guidelines. Only studies involving adult human samples were included. Dysregulated miRNAs were standardized using miRBase and analyzed with miRNet v2.0, miRTarBase v9.0, and DIANA-miRPath v3.0. Interaction networks were constructed in Cytoscape, and functional enrichment analyses were performed using ClusterProfiler and MSigDB to identify shared pathways and gene targets.

**Results:**

The analysis revealed a set of miRNAs altered in both TB and T2DM, including hsa-miR-21, hsa-miR-29a-3p, hsa-miR-125a-5p, hsa-miR-125b, hsa-miR-130b, hsa-miR-144, hsa-miR-155, hsa-miR-223, and hsa-miR-486. These miRNAs converge on central target genes such as STAT3, PTEN, BCL2, MYC, RAF1, EGFR, IRS1, SMAD4, FOXO3, GLUT4, AKT1, and CTNNB1, regulating pathways of insulin signaling, glucose metabolism, apoptosis, inflammation, and fibrosis.

**Discussion:**

Shared miRNAs act as molecular nodes linking immunity and metabolism, providing a framework for biomarker development in TB-T2DM comorbidity. Their regulatory convergence suggests potential applications in diagnosis, prognosis, and therapeutic innovation, particularly in vulnerable populations where both diseases intersect. These findings underscore the importance of integrating immunometabolic biomarkers into personalized medicine strategies to address the dual burden of TB and T2DM.

## Introduction

Tuberculosis (TB) and type 2 diabetes mellitus (T2DM) represent two of the greatest public health challenges worldwide, due to their high prevalence in low- and middle-income countries (LMICs) and their substantial clinical and socioeconomic impact. TB, caused by Mycobacterium tuberculosis (Mtb), continues to be one of the leading causes of infectious morbidity and mortality globally, with more than 10 million new cases and 1.3 million deaths reported annually (1). On the other hand, T2DM has reached epidemic proportions, with approximately 537 million adults (20–79 years) living with diabetes globally in 2021, projected to reach 643 million by 2030 and 783 million by 2045 (2). The burden of both diseases is particularly high in LMICs, where TB is endemic.

The coexistence of TB and T2DM in a single patient presents significant clinical challenges. Patients with T2DM have a threefold increased risk of developing active TB, in addition to more severe clinical presentations, including higher bacillary burden, poor response or resistance to treatment, delayed culture conversion, and higher probability of relapse (3, 4). Hyperglycemia alters the function of macrophages and T lymphocytes, dysregulates cytokine production (often reducing proinflammatory mediators while increasing anti-inflammatory cytokines), and impairs the formation and maintenance of granulomas multicellular structures essential for infection control (5). In this context, there is an urgent need to identify molecular biomarkers that reflect the convergence of immunological and metabolic processes in TB-T2DM comorbidity.

MicroRNAs (miRNAs) are key regulators of post-transcriptional gene expression involved in both TB and T2DM. These small non-coding molecules, approximately 22 nucleotides in length, modulate critical pathways such as lymphocyte differentiation, macrophage polarization, cytokine signaling, and cellular metabolism (6). Altered miRNA expression profiles have been reported in multiple infectious and metabolic diseases, including TB and T2DM, suggesting a central role in shared pathophysiology (7, 8).

In TB, several miRNAs, such as hsa-miR-21, hsa-miR-29a, hsa-miR-223, and hsa-miR-155, have been associated with regulation of innate and adaptive immune responses. For example, hsa-miR-21 has been linked to suppression of Th1 responses, while hsa-miR-29a regulates expression of genes involved in cell adhesion and extracellular matrix remodeling (9, 10). hsa-miR-223 modulates FOXO3 expression, affecting apoptosis of infected macrophages (11), and hsa-miR-155 is involved in regulation of macrophage polarization and cytokine production (12).

In T2DM, miRNAs such as hsa-miR-144, hsa-miR-126, and hsa-miR-130b are linked to insulin resistance, pancreatic beta cell dysfunction, and microvascular complications. hsa-miR-144 decreases IRS1 expression, affecting insulin signaling (13) hsa-miR-126 is associated with vascular complications and diabetic retinopathy (14), and hsa-miR-130b regulates processes of lipid metabolism and insulin secretion (15, 16). These altered expression profiles reflect the complexity of post-transcriptional regulation in T2DM and its impact on metabolic homeostasis.

Specific miRNAs are dysregulated in both pathologies and may act as common molecular nodes integrating immune and metabolic responses. Among them are hsa-miR-21, hsa-miR-29a, hsa-miR-223, hsa-miR-144, and hsa-miR-155, which are involved in both immunity against Mtb and regulation of insulin signaling and cellular metabolism. The identification of these shared miRNAs represents a fundamental finding suggesting the existence of convergent molecular mechanisms that could explain, at least partially, the increased susceptibility to TB observed in patients with diabetes (5, 17).

Despite growing evidence on the role of miRNAs in TB and T2DM, there is limited literature reporting miRNA activity in this comorbidity. To our knowledge, this is the first systematic review with comprehensive bioinformatic analysis to identify and characterize miRNAs shared between both diseases. By integrating data from reference databases such as miRNet, miRTarBase, and DIANA-miRPath, we constructed networks of miRNA-mRNA interactions that reflect convergent molecular mechanisms in both pathologies.

The specific aims of this study are to:

1. Identify miRNAs reported as dysregulated in pulmonary TB and T2DM in experimental studies with human samples.
2. Compare expression profiles to determine miRNAs shared between both pathologies.
3. Analyze miRNA-mRNA interactions using bioinformatics tools, identifying experimentally validated target genes and enriched biological pathways.
4. Generate evidence for the potential application of miRNAs as diagnostic and prognostic biomarkers in TB-T2DM comorbidity.

TB-T2DM comorbidity represents a growing challenge for health systems, particularly in low- and middle-income countries (LMICs) such as Mexico, India, and China, where the prevalence of T2DM is increasing and TB remains endemic. The identification of shared molecular biomarkers will enable progress toward personalized medicine, capable of stratifying patients according to their risk of developing active TB, optimizing therapeutic regimens, and reducing complications. This approach contributes to understanding the immunometabolic mechanisms underlying the interaction between chronic infections and metabolic diseases, providing evidence for the design of new prevention and treatment strategies.

## Methodology

### Search strategy

A systematic search was carried out in the PubMed and Google Scholar databases, using *Medical Subject Headings* (MeSH) terms and keywords related *to “microRNA”*, “*dysregulated expression*”, “*biomarkers*”, *“type 2 diabetes mellitus”* and *“tuberculosis”*. The search included articles published from October 2011 to July 2025 in English. Potentially relevant publications were identified through a manual review of titles and abstracts.

### Selection criteria

Studies were eligible if they 1) reported miRNA expression profiles in adult human samples related to pulmonary TB or T2DM; 2) specified the biological sample type and study groups; 3) described the methodology used to quantify miRNAs; and 4) clearly reported the differential expression of miRNAs. We excluded systematic reviews and meta-analyses without experimental validation, studies lacking a description of the miRNA detection methodology, and experimental studies conducted in children, animal models, or cell lines.

### Quality assessment

**Figure 1:**
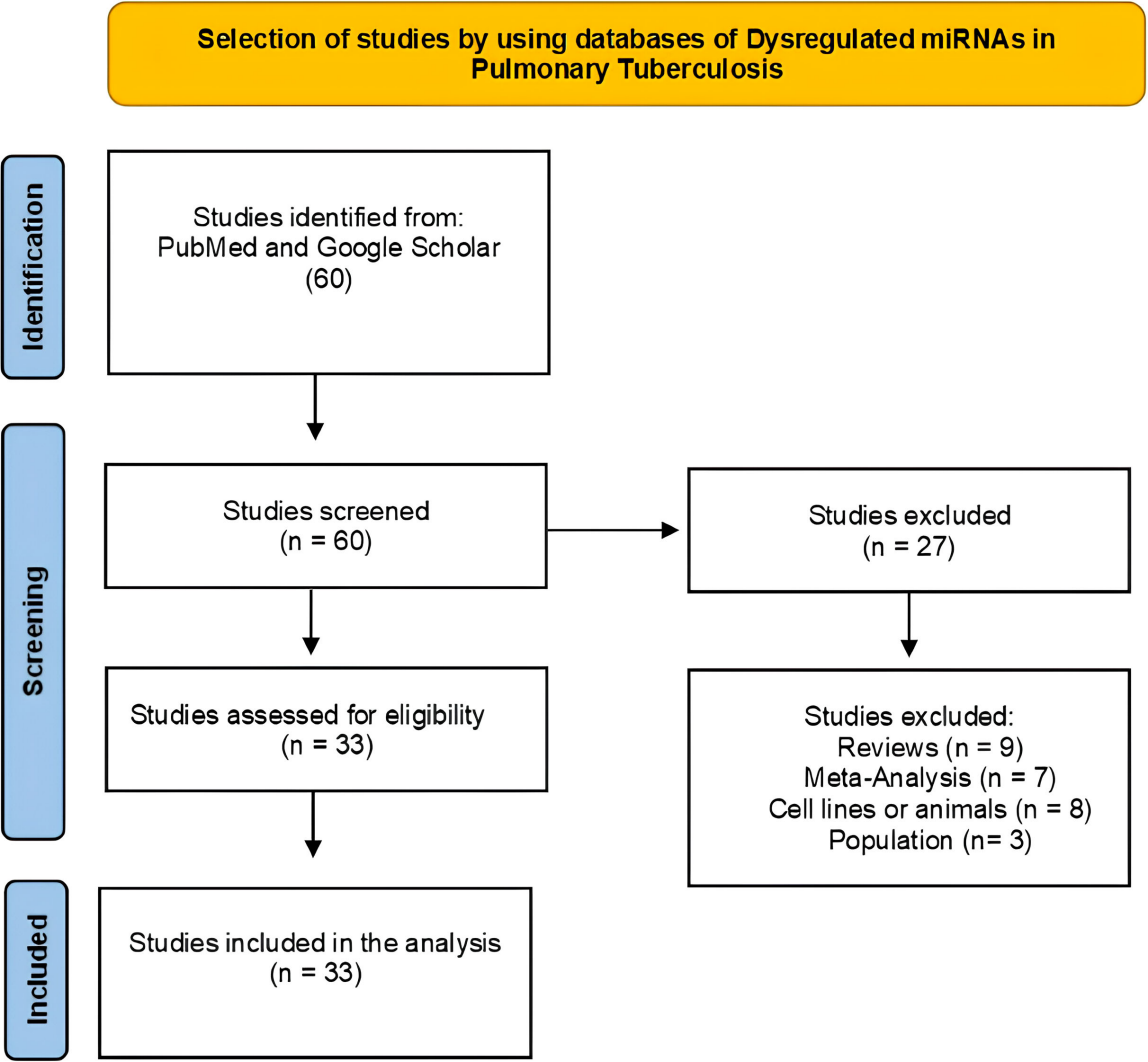
Flowchart from the systematic review of studies selected to identify dysregulated miRNAs in pulmonary TB. Made with PRISMA 2020-compliant flow diagrams.

**Figure 2:**
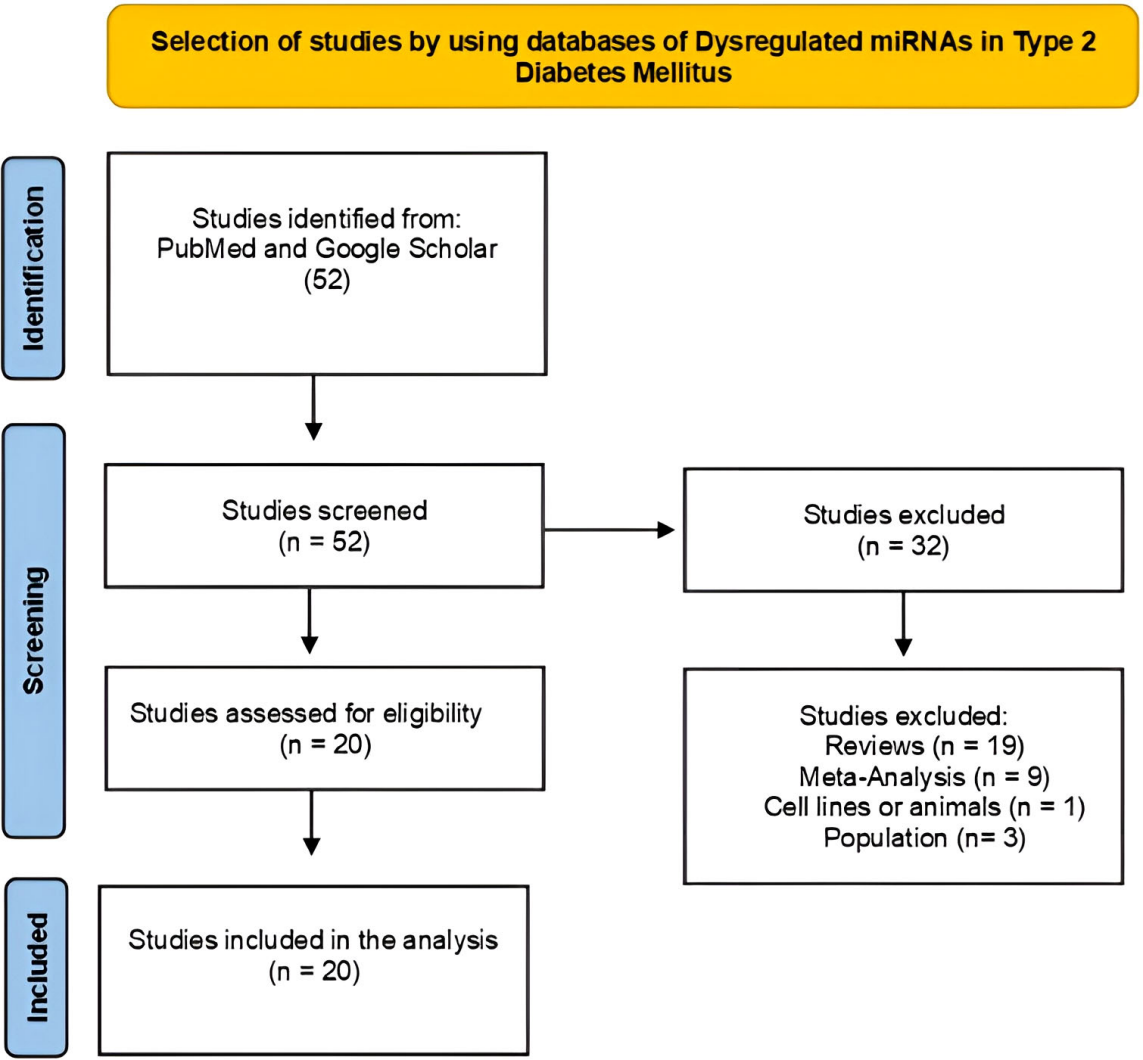
Flowchart of the systematic review of the studies selected to identify dysregulated miRNAs in DM2. Made with PRISMA 2020-compliant flow diagrams.

The systematic review followed the PRISMA 2020 guidelines, and flow diagrams were used to document the identification, screening, eligibility, and inclusion of studies for each condition (**Figures 1**, **2**). A comprehensive screening of titles, abstracts, and full texts was performed. We did not apply a formal risk of bias assessment tool. A total of 60 articles were evaluated to identify dysregulated miRNAs in pulmonary TB, of which 27 were excluded because they did not meet the selection criteria (**Figure 1**). In the case of miRNAs selected for DM2, 52 publications were analyzed, and 32 were discarded for the same reason (**Figure 2**).

### Selection of miRNAs with an altered expression profile for both pathologies

In the selection of miRNAs, a review flow was systematically followed, which consisted of 1) review of titles and abstracts, 2) reading of the full text of the articles, and 3) elaboration of a miRNA table for each disease (**Tables 1**, **2**). The names of the miRNAs were standardized using the **miRBase database.** Subsequently, a comparative table was constructed that included only the dysregulated miRNAs present in both diseases (**Table 3**).

**Table 1:** Selection of dysregulated miRNAs in pulmonary TB, according to the literature consulted.

Author	MicroRNA Name	Pulmonary Tuberculosis			
		Function / Utility	Methodology	Sample	Country / Population
Wang C et al., (18)	hsa-miR-130b hsa-miR-21 hsa-miR-223 hsa-miR-302a hsa-miR-424 hsa-miR-451 hsa-miR-486-5p hsa-miR-144 hsa-miR-133a hsa-miR-365 hsa-miR-424 hsa-miR-500 hsa-miR-661 hsa-miR-892b	Affects the development of immune cells, promoting the pathogenesis of TB	Micro-Array. RT-qPCR (TaqMan).	Peripheral blood (PBMC)	China Adults (35 – 45 years)
Latorre I et al., (19)	hsa-miR-21 hsa-miR-29c hsa-miR-194 hsa-miR-150	Reduction of host Th1 cell responses.	Micro-Array. RT-qPCR (TaqMan).	Peripheral blood (PBMC)	Spain Adults (> 18 years)
Zheng L et al., (20)	hsa-miR-16 hsa-miR-137 hsa-miR-140-3p hsa-miR-193a-3p hsa-miR-501-5p hsa-miR-598 hsa-miR-95 hsa-miR-101 hsa-miR-150	Activation of immunoregulatory interactions involved with the TGF-β signaling pathway and the T cell receptor signaling pathway.	Micro-Array. RT-qPCR (TaqMan).	Peripheral blood (PBMC)	Hong Kong Adults (> 18 years)
Song Q et al., (21)	hsa-miR-365	Regulation the expression of IL-6 by binding to its 3 ´UTR, modulating the immune response against ATB.	RT-qPCR. Western Blot. ELISA.	Bronchoalveolar macrophages, serum and whole blood (PBMC)	China Adults (> 18 years)
Liu Y et al., (22)	hsa-miR-582-5p	Inhibition of monocyte apoptosis in ATB patients by negatively regulating FOXO1 expression.	RT-qPCR. Apoptosis assays by FOXO1 transfection.	Peripheral blood (monocytes)	China Adults (20 - 60 years)
Xi X et al., (11)	hsa-miR-223	Regulation of the immune response against *Mtb* by modulation of FOXO3 expression.	RT-qPCR. Apoptosis assays by FOXO3 transfection.	Peripheral blood (PBMC/macrophages)	China Adults (29 - 60 years)
Zhang Y et al., (23)	hsa-miR-892b hsa-miR-199b-5p hsa-miR-582–5p	Regulation of the MAPK signaling pathway and leukocyte migration.	RT-qPCR.	Peripheral blood (PBMC)	China Adults (> 18 years)
Alipoor SD et al., (24)	hsa-miR-484 hsa-miR-425 hsa-miR-96	Modulation of catabolism and cellular metabolism, inhibition of the innate immune response and promotes the dissemination of *Mtb*	RT-qPCR.	Serum (exosomes)	Iran Adults (18-65 years)
Chakrabarty S et al., (25)	hsa-miR-146a-5p hsa-miR-486-5p hsa-miR-16-2-3p hsa-miR-144-3p hsa-miR-483-5p hsa-miR-193a-5p hsa-miR-140-3p hsa-miR-125b-5p hsa-miR-425-5p hsa-miR-101-3p hsa-miR-29c-3p hsa-miR-378a-5p hsa-miR-143-3p hsa-miR-378a-3p hsa-miR-378d hsa-miR-378f hsa-miR-378c hsa-miR-29c-5p hsa-miR-22-3p hsa-miR-320a hsa-miR-129-5p hsa-miR-409-3p hsa-miR-370-3p hsa-miR-551b-5p hsa-miR-4523 has-mir-425-5p hsa-miR-4523	Differentially expressed in TB patients, they modulate pathways involved with cell cycle, transcriptional regulation, apoptosis, mRNA processing, lipid metabolism, and cell-cell communication.	RT-qPCR.	Serum (RNA)	India Adults (30 – 65 years)
Massi MN et al., (26)	has-mir-425-5p hsa-miR-4523	Differential diagnosis of PTB and LTBI. hsa-mir-425-5p is positively regulated and hsa-mir-4523 is negatively regulated in PTB.	RT-qPCR.	Whole blood (RNA)	Indonesia Adults (25 – 60 years)
Adhikary S et al., (27)	hsa-miR-29a	Differential diagnosis of extrapulmonary TB and pulmonary TB.	RT-qPCR.	Serum (RNA)	India Adults (18 - 76 years)
Hurtado, J et al., (28)	hsa-miR-126-3p hsa-miR-378a-3p hsa-miR-423-5p	Monitoring of severe cases of TB.	Sequencing (ILLUMINA) RT-q-PCR	Serum (RNA)	Uruguay Adults (> 18 years)
El-Masry et al., (29)	hsa-miR-21-5p hsa-miR-29a-3P hsa-miR-361-5p	Diagnostic and prognostic markers of ATB and LTBI in humans.	RT-qPCR.	Whole blood (RNA)	Egypt Adults (> 18 years)
Kim, J et al., (30)	hsa-miR199a-3p hsa-miR199b-3p hsa-miR-6856-3p hsa-miR-374c-5p hsa-miR-16-5p	Promising biomarkers for the diagnosis and therapeutic monitoring of ATB.	Sequencing (ILLUMINA) RT-q-PCR	Serum (RNA)	Korea Adults (35 - 43 years)
Sun X et al., (31)	hsa-miR-125b	Decreases levels of IL-6, TNF-α, NF-κB and IFN-γ by inhibiting RAF1	RT-qPCR (TaqMan)	Peripheral blood (PBMC)	China Adults (> 18 years)
Ndzi EN et al., (32)	hsa-miR-29a-3p hsa-miR-155-5p hsa-miR-29a-3p	Biomarkers useful for differentiating pulmonary TB from extrapulmonary TB.	RT-qPCR (TaqMan)	Peripheral blood (RNA)	Africa Adults (> 18 years)
Barry SE et al., (10)	hsa-miR-99b hsa-miR-29a hsa-miR-146a hsa-miR-26a hsa-miR -652	Biomarker profile with potential to predict treatment response.	RT-qPCR (TaqMan)	Plasma (RNA)	China Adults (18 – 69 years)
Corral-Fernández et al., (33)	hsa-miR-326	Modulation of the immune response induced by antituberculosis treatment.	RT-qPCR (TaqMan)	Peripheral blood (PBMC)	Mexico Adults (30 – 55 years)
Wang C et al., (34)	hsa-miR-21-5p hsa-miR-92a-3p hsa-miR-148b-3p hsa-miR-125a-5p	Potential biomarkers for monitoring treatment in ATB patients	RT-qPCR (TaqMan)	Plasma (RNA)	China Adults (18 – 65 years)
Cui JY et al., (35)	hsa-miR-769-5p hsa-miR-320a hsa-miR-22-3p	Non-invasive biomarkers for the diagnosis and prognosis of ATB	RT-qPCR (TaqMan)	Plasma (RNA)	China Adults (30 – 60 years)
Wang C et al., (36)	hsa-miR-365 hsa-miR-29a hsa-miR-16 hsa-miR-155	Non-invasive biomarkers for early diagnosis and prognostic treatment of ATB	RT-qPCR (TaqMan)	Peripheral blood (PBMC)	China Adults (19 – 77 years)
Carranza et al., (37)	hsa-miR-let-7e-5p hsa-miR-197-3p hsa-miR-223-3p	Possible biomarker of therapeutic failure or relapse in DR-TB	Micro-Array. RT-qPCR (TaqMan)	Serum (Exosomes)	Mexico Adults (18 to 77 years)
Shepelkova G et al., (12)	hsa-miR-191 hsa-miR-223 hsa-miR-155	Biomarker for monitoring treatment and lung destruction	RT-qPCR (TaqMan)	Serum (RNA)	Russia Adults (18 – 65 years)
Sun W et al., (38)	hsa-miR-140-5p	Negative regulation of the SNHG16 gene favoring the control of *Mtb* infection.	RT-qPCR (TaqMan)	Peripheral blood (PBMC)	China Adults (30 – 40 years)
Liu G et al., (39)	hsa-miR-125b-5p	Decreased inflammation and increased apoptosis, reducing bacterial load, through negative regulation of DRAM2.	RT-qPCR (TaqMan)	Peripheral blood (PBMC)	China Adults (25 – 65 years)
Tu H et al., (40)	hsa-miR-423-5p hsa-miR-17-5p hsa-miR-20b-5p	Inhibition of autophagosome-lysosome fusion by post-transcriptional regulation of VPS33A, favoring *Mtb* replication.	RT-qPCR (TaqMan)	Serum (RNA) Peripheral blood (PBMC)	China Adults (25 – 55 years)
Yang, J. et al., (41)	hsa-miR-15a- 5p	Regulation of key cell cycle genes (CCND1, CDK6, CCND2). Plays a central role in TB pathogenesis.	Sequencing RT-q-PCR	Plasma (RNA)	China Adults (28 – 45 years)
Cui, J et al., (42)	hsa-miR-766-3p	Potential diagnostic biomarker and key modulator of the cellular response to *Mtb* infection by suppressing NRAMP1.	RT-qPCR (TaqMan)	Plasma (Exosomes)	China Adults (20 – 55 years)
Liu J et al., (43)	hsa-miR-Let-7d-5p hsa-miR-140-5p	Diagnostic biomarker to differentiate ATB from LTBI.	RT-qPCR (TaqMan)	Serum (RNA)	China Adults (18 – 80 years)
Sampath P et al., (44)	hsa-miR-548m hsa-miR-486-3p HSA-MIR-486-3P hsa-miR-132-3p hsa miR-150-5p	Alterations in the expression profile of this panel are associated with immune dysfunction, drug resistance, and severe cases of TB.	Flow cytometry NanoString	Peripheral blood (PBMC)	India Adults (18 - 47 years)
Zhang H et al., (45)	hsa-miR-196b hsa-miR-376c	Regulation of the immune response against *Mtb*. Diagnostic biomarkers of ATB.	RT-qPCR (TaqMan)	Serum (RNA)	China Adults (18 – 45 years)
Fu, Y et al., (9)	hsa-miR-29a hsa-miR-93 hsa-miR-3125	Potential diagnostic biomarker panel in ATB.	Micro-Array. RT-qPCR (TaqMan)	Serum (RNA) Sputum (RNA)	China Adults (18 – 45 years)
Spinelli, S. et al., (46)	has-miR-424 has-miR-146a has-miR-223 has-miR-144 has-miR-421	Modulation of inflammatory response. Monitoring of *anti-Mtb* therapy.	RT-qPCR (TaqMan)	Peripheral blood (PBMC)	Argentina Adults (18 – 71 years)

**Table 2:** Selection of dysregulated miRNAs in DM2, according to the literature consulted.

Author	MicroRNA Name	Type 2 Diabetes Mellitus			
		Function / Utility	Methodology	Sample	Country / Population
Karolina DS et al., (13)	hsa-miR-144	Affects insulin signaling by decreasing IRS1 expression	RT-qPCR (TaqMan)	Peripheral blood (PBMC)	Singapore Adults (21 – 71 years)
Kameswaran V et al., (47)	hsa-miR-7-3-5p	Regulation of insulin secretion and proliferation of pancreatic beta cells.	RNA-Seq RT-q-PCR	Pancreatic beta cell islets (autopsy)	USED Adults (45 – 61 years)
Fuentevilla-Alvarez G et al., (48)	hsa-miR-17-5p	Regulation of proliferation, migration, and angiogenesis in endothelial cells.	RT-qPCR (TaqMan)	Peripheral blood (PBMC)	Mexico Adults (> 40 years)
Sulaiman F et al., (16)	hsa-miR-130b-5p hsa-miR-130b-3p hsa-miR-125a-3p hsa-miR-223-5p hsa-miR-143-3p hsa-miR-29a-3p	Related to lipid metabolism and elevated cholesterol levels. Pancreatic beta cell dysfunction. Insulin resistance. Impaired insulin secretion.	RNA-Seq	Peripheral blood (PBMC)	Dubai Adults (18 – 87 years)
Ofori et al., (15)	hsa-miR-130b-3p hsa-miR-130a-3p hsa-miR-152-3p	Failure of insulin production in pancreatic beta cells.	RT-qPCR (TaqMan)	Pancreatic beta cell islets (autopsy)	Sweden Adults (> 18 years)
Katayama M et al., (49)	hsa-miR-20b-5p	Increased glycogen synthesis in skeletal muscle cells by the regulation of AKTIP and STAT3	RT-qPCR (TaqMan)	Peripheral blood (PBMC)	Sweden Adults (> 18 years)
Shaker OG et al., (50)	hsa-miR-20b hsa-miR-17-3p	Negative correlation with fasting blood glucose, triglycerides, and glycosylated hemoglobin.	RT-qPCR (TaqMan)	Peripheral blood (PBMC)	Egypt Adults (49 – 55 years)
Weale CJ et al., (51)	hsa-miR-1299 hsa-miR-126-3p hsa-miR-30e-3p	Associated with prediabetes, diabetes, and micro- and macrovascular complications. Negative correlation with abdominal circumference. Positive correlation with glucose and HDL cholesterol levels.	RT-qPCR (TaqMan)	Peripheral blood (RNA)	South Africa Adults (> 45 years)
Roux M et al., (52)	hsa-miR-152-3p	Associated with diabetic nephropathy and loss of plasma osmolarity due to silencing of the SLC5A3 gene.	RT-qPCR (TaqMan)	Peripheral blood (RNA)	France Adults (> 18 years)
Ali H et al., (53)	hsa-miR-151a-3p hsa-miR-182-5p	Related to cell growth and differentiation, glucose and lipid metabolism in patients with DM2 and diabetic nephropathy. Modulation of gene expression (PTEN, SMAD2, SMAD4, VEGF, CCND2, CDK6, LIN28B, and CHD1).	RNA-Seq	Urine (extracellular vesicles)	Asia Adults (> 18 years)
Greco M et al., (54)	hsa-miR-1281	Increased VEGFA expression. Related to diabetic retinopathy.	RT-qPCR (TaqMan)	Peripheral blood (RNA)	Italy Adults (18 -60 years)
Syed TR et al., (55)	hsa-miR-126 hsa-miR-486 hsa-miR-223 hsa-miR-375	Inhibition of the insulin receptors IRS1 and GLUT4. Promotes insulin resistance. Associated with elevated levels of fasting blood glucose and glycosylated hemoglobin.	RT-qPCR (TaqMan)	Peripheral blood (RNA)	India Adults (< 45 years)
Yang T. T. et al., (56)	hsa-miR-155	Involved in the pathogenesis of T2DM retinopathy by regulating Treg cells with TGF-β.	RT-qPCR (TaqMan)	Peripheral blood (RNA)	China Adults (37 – 78 years)
Xu Bai et al., (57)	hsa-miR-155	Potential biomarker of retinopathy and nephropathy in patients with T2DM	RT-qPCR (TaqMan)	Peripheral blood and urine (RNA).	China Adults (63 – 73 years)
Guglielmi V et al., (58)	hsa-miR-21	Related to insulin resistance and progression of obesity	RT-qPCR (TaqMan)	Subcutaneous adipocytes (RNA)	Italy Adults (> 40 years)
McClelland A. et al., (59)	hsa-miR-21	Related to the development of kidney disease and fibrosis in patients with T2DM.	RT-qPCR (TaqMan)	Renal biopsy (RNA)	Israel Adults
Meng X et al., (60)	hsa- miR23a-3p	Association with severe kidney disease in patients with T2DM	RT-qPCR (TaqMan)	Serum (RNA).	China Adults (> 30 years)
Lozano-Bartolomé J et al., (61)	hsa-miR23a-3p hsa-miR181a-5p	Negative correlation with insulin resistance	RT-qPCR (TaqMan)	Adipose tissue (RNA) Serum (RNA)	Spain Adults (45 – 55 years
Fang Y et al., (62)	hsa-miR183a-5p hsa-miR125a-5p	Association with insulin signaling and glucose transport.	RT-qPCR (TaqMan)	Urine	China Adults (> 30 years)
Zhang T et al., (14)	hsa-miR-126	Decreased miR-126 may be an early marker of T2DM.	RT-qPCR (TaqMan)	Plasma (RNA).	China Adults (> 30 years)

**Table 3:** dysregulated miRNAs in both conditions (pulmonary TB and DM2).

microRNA name	Expression	
	Pulmonary tuberculosis	Type 2 diabetes mellitus
hsa-miR-21	Up	Up
hsa-miR-125a-5p	Up	Up
hsa-miR-486	Up	Down
hsa-miR-223	Up	Up
	Down	Down
hsa-miR-144	Up	Up
hsa-miR-130b-5p	Down	Down
hsa-miR-125a-3p	Up	Up
hsa-miR-223-5p	Up	Up
hsa-miR-29a-3p	Up	Up
hsa-miR-130b-3p	Down	Up
hsa-miR-130a-3p	Up	Up
hsa-miR-155	Up	Up

### Bioinformatics analysis

Once the dysregulated miRNAs in DM2 and TB were identified, the list of target mRNAs was obtained. To do this, the miRNet v2.0 (https://www.mirnet.ca/miRNet/home.xhtml) database was consulted, loading the list of overexpressed, underexpressed, and variable expression miRNAs in both diseases. Subsequently, from each list of miRNAs, the mRNAs that have been experimentally validated and reported in the miRTarBase v9.0 (https://mirtarbase.cuhk.edu.cn/~miRTarBase/miRTarBase_2025/php/index.php) database were obtained. The significant interactions between miRNAs-mRNAs obtained to build the network using Cytoscape v3.10.3 were obtained. The CytoHubba v0.1 application was used to obtain the most central genes of the miRNA-mRNA network, based on the value of the percolated component of bonds (EPC).

To identify the functional annotation of the miRNAs and target genes, a pathway analysis was performed using the R v4.5.0 programming language with the ClusterProfiler v4.16.0 (63) and the DIANA-miRPath v3.0 platform (https://dianalab.e-ce.uth.gr/html/mirpathv3/index.php?r=mirpath) (64). For the R-based analysis, the list of target mRNAs obtained from miRNet was used, and gene sets from the Molecular Signatures Database (MSigDB) were downloaded with the msigdbr v25.1.0 package, retaining only those pathways relevant to the immune response against tuberculosis. For the DIANA-miRPath v3.0 analysis, the names of the shared miRNAs were uploaded, and pathway enrichment was evaluated using Gene Ontology (GO) biological process terms.

## Results

### Identification of dysregulated miRNAs shared between tuberculosis and type 2 diabetes mellitus

The systematic review revealed miRNAs with comparable expression changes in TB and T2DM patients. They were grouped into three categories: overexpressed, underexpressed, and variably expressed, highlighting the heterogeneity reported across studies.

Identifying these shared miRNAs suggests convergent molecular mechanisms that may partly explain the heightened TB susceptibility in diabetic patients (65–67).

### Analysis of miRNAs-mRNA interaction networks and identification of target genes

**Figure 3:**
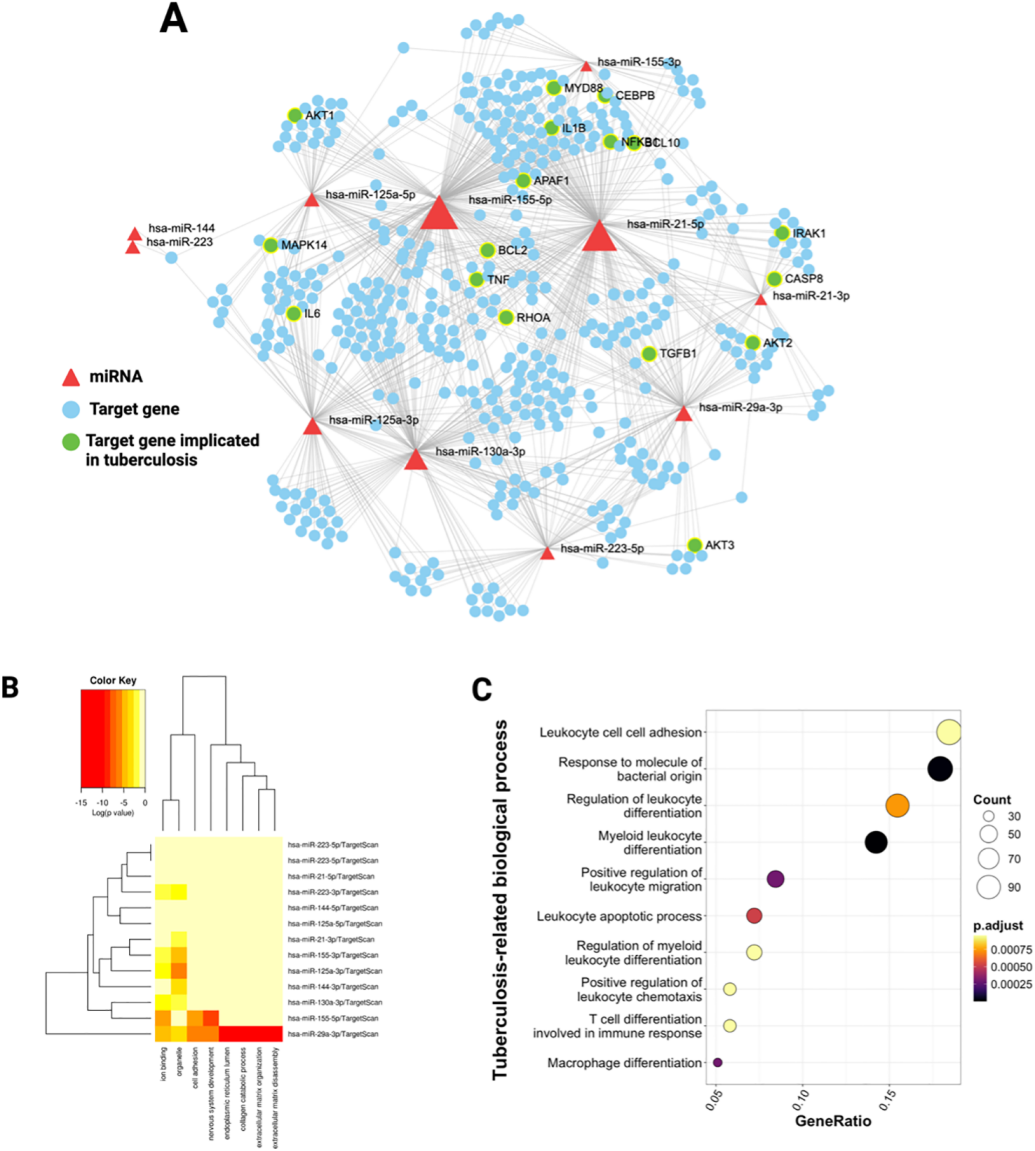
MiRNAs over-expressed in T2DM and TB regulate the innate immune response to tuberculosis. **(A)** Target miRNA-mRNA interaction network obtained from the list of over-expressed and shared miRNAs between TB and T2DM. The network contains the target mRNAs of the miRNAs, experimentally validated and deposited in the miRTarbase database. The red triangles show the miRNAs and the white mRNAs in circles. White mRNAs related to the TB response are highlighted in green circles. **(B)** Pathways regulated by overexpressed miRNAs in TB and T2DM. The heat map shows the significantly enriched pathways from the GO database. The color intensity represents the p-value corrected by FDR. **(C)** Biological processes of the immune response to tuberculosis significantly enriched in target mRNAs. The size of the dots represents the number of white mRNAs present in each pathway. The color of the dots shows the set p-value.

**Figure 4:**
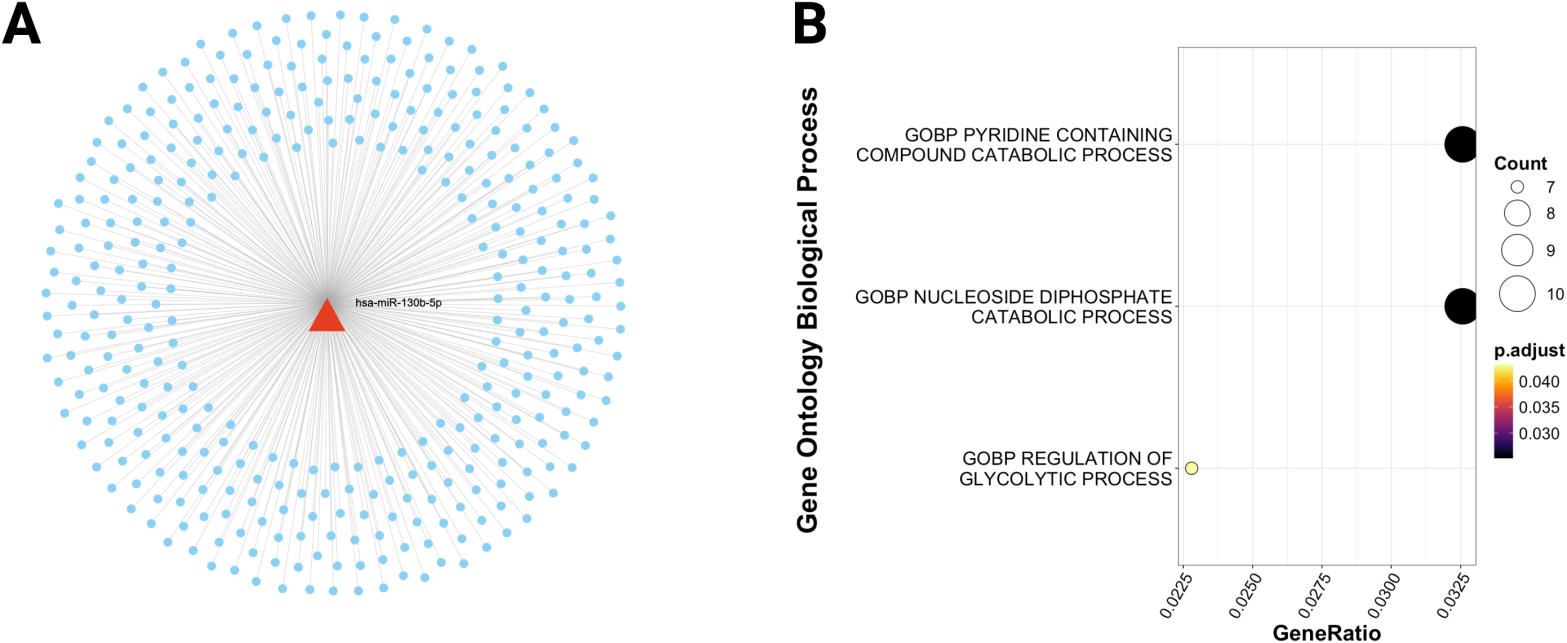
MiRNAs with decreased expression regulate glucose and nucleotide metabolic processes. **(A)** Target miRNA-mRNA interaction network obtained from the list of underexpressed and shared miRNAs between TB and T2DM. **(B)** Biological processes enriched in the target mRNAs of underexpressed miRNAs. The size of the dots represents the number of white mRNAs present in each pathway. The color of the dots shows the set p-value.

**Figure 5:**
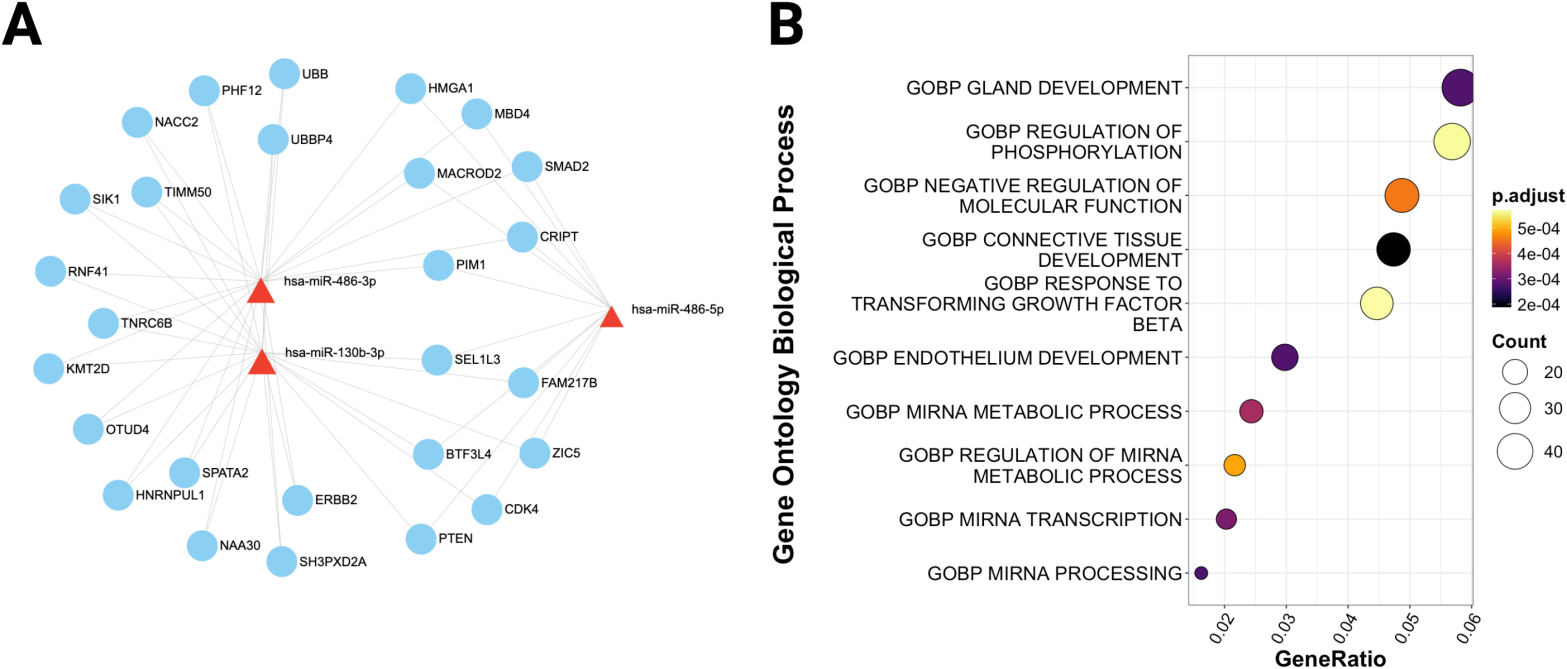
MiRNAs with variable expression impact the development of specialized tissues and glands. **(A)** Target miRNA-mRNA interaction network obtained from the list of miRNAs with variable expression between TB and T2DM. **(B)** Biological processes enriched in the target mRNAs of the miRNAs. The size of the dots represents the number of white mRNAs present in each pathway. The color of the dots shows the set p-value.

To understand the functional implications of the shared miRNAs, we queried the miRNet v2.0 database with the list of dysregulated miRNAs to retrieve their target mRNAs. The analysis uncovered a network of interactions, including 2,887 targets for overexpressed miRNAs, 355 for underexpressed, and 853 for variably expressed. (**Figures 3A**, **4A**, **5A**). This network indicates that shared miRNAs influence diverse intracellular pathways, modulating immune, metabolic, and signaling processes essential for host defense against Mtb (17, 68, 69). The differential in the number of target genes identified for each category of miRNAs reflects the regulatory complexity inherent in these non-coding RNAs. Overexpressed miRNAs more effectively repress target mRNAs, accounting for the larger number of targets and their wider impact on cell physiology (70–72).

For underexpressed miRNAs, other mechanisms, such as long non-coding RNAs (lncRNAs), may influence their levels. For example, lncRNAs act as sponges for miRNAs, decreasing their expression (73).

### Functional enrichment analysis: biological processes regulated by shared MiRNAs

#### Overexpressed miRNAs: suppression of essential immune processes

The biological pathway enrichment analysis performed on the DIANA-miRPath platform revealed that overexpressed miRNAs in both pathologies regulate pathways involved in extracellular matrix remodeling and organization.

Specifically, hsa-miR-29a-3p is implicated in collagen catabolism and cell adhesion in the extracellular matrix (**Figure 3B**). *In vitro* studies carried out on endothelial cells from different tissues have shown that the overexpression of hsa-miR-29a-3p decreases the expression of adhesion molecules such as VCAM-1, ICAM-1, and E-Selectin (74). These results suggest that miRNAs regulate genes associated with extracellular matrix organization, thereby influencing cell adhesion. On the other hand, pathway analysis focused on the target mRNAs of overexpressed miRNAs revealed that there is a downregulation of the expression of genes involved in immunological processes critical for the defense against tuberculosis, including leukocyte adhesion, differentiation, and migration, as well as the tuberculosis-specific immune response (**Figure 3C**). This finding is pathophysiologically relevant, as the overexpression of these shared miRNAs may suppress host defense mechanisms, facilitating latent TB reactivation in diabetic patients and progression of active disease (65–67). Leukocyte adhesion and migration are essential for immune cell recruitment to infection sites and for granuloma development and stability (75–77).

The alteration of these processes in the context of TB-T2DM comorbidity has been documented in transcriptomic studies, where alterations in cell trafficking and granuloma architecture are observed, potentially contributing to increased bacillary load and reduced immune efficacy in diabetic patients (66, 67, 78, 79).

Leukocyte differentiation, particularly macrophage polarization toward M1 or M2 phenotypes, influences the balance between bacterial control and tissue immunopathology (65, 79, 80).

Hyperglycemia in DM2 alters macrophage polarization, promoting phenotypes less effective in Mtb clearance and antigen presentation (65, 78). The overexpression of miRNAs targeting genes involved in leukocyte differentiation may sustain immune dysfunction, impairing mycobacterial clearance (81, 82).

### Underexpressed and variably expressed miRNAs: regulation of cell metabolism

In contrast to overexpressed miRNAs, pathway analysis of underexpressed or variably expressed miRNAs in the context of the immune response to tuberculosis did not yield statistically significant results.

Nevertheless, functional enrichment analysis using the Gene Ontology Biological Process database indicated that mRNAs regulated by these miRNAs participate in key metabolic processes, including nitrogenous base metabolism, glucose metabolism, miRNA biogenesis, and the development of specialized tissues such as endocrine glands and vascular endothelium (**Figures 4B**, **5B**).

These findings suggest that underexpressed and variably expressed miRNAs may contribute to the pathophysiology of TB–T2DM comorbidity by modulating metabolic pathways that, while not directly linked to the innate immune response against tuberculosis, are essential for maintaining cellular and tissue homeostasis under metabolic and infectious stress (66, 79). For instance, glucose metabolism is critical for both immune cell activity and glycemic control in T2DM, and its dysregulation can worsen both conditions (66, 83). Moreover, TB has been reported to impair the function of glands such as lymph nodes, adrenal glands, and the thyroid (84, 85), leading to endocrine disturbances that influence immune activation (86).

Because subsequent analyses aimed to clarify immunological mechanisms directly associated with tuberculosis susceptibility and progression in the context of diabetes, we concentrated on overexpressed miRNAs, whose functional impact on the immune response was more evident and statistically significant.

### Identification of core genes (hub genes) in the miRNA-mRNA network

**Figure 6:**
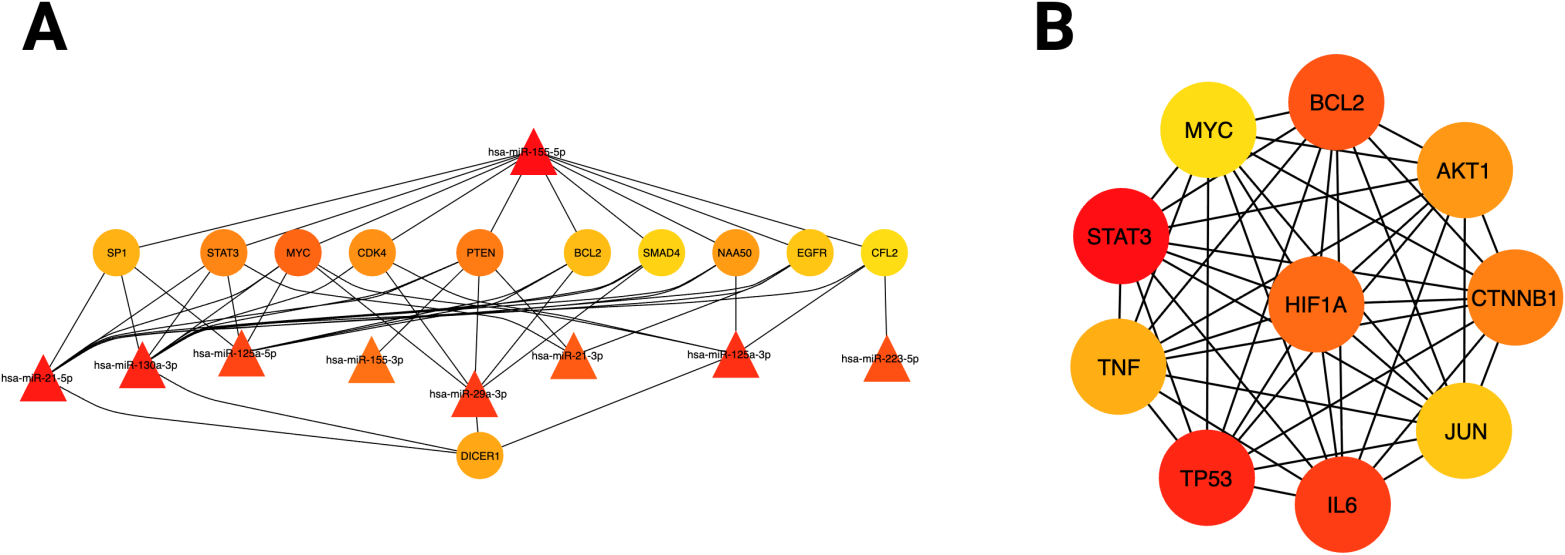
Identification of hub genes in miRNA-mRNA interaction networks. **(A)** Analysis of the hub genes in the integrated miRNA-mRNA network obtained from miRNet for over-expressed miRNAs. **(B)** mRNA network where the hub genes derived from topological analysis are highlighted. The color intensity represents the value of the percolated bond component (EPC) of each node.

To explore the functional interactions between overexpressed miRNAs and their target mRNAs, a molecular interaction network was constructed using experimentally validated targets from the miRTarBase v9.0 database. Work centrality analysis, with the CytoHubba application, identified 11 mRNAs with high centrality (hub genes), representing key nodes in the regulatory network: *DICER1, SP1, STAT3, MYC, CDK4, PTEN, BCL2, SMAD4, NAA50, EGFR*, and *CFL2* (**Figure 6A**).

These hub genes have pleiotropic roles in regulating fundamental cellular processes. DICER1, a ribonuclease essential for miRNA biogenesis, functions as a central regulator of the RNA interference system, and modulation by miRNAs may establish feedback loops that amplify or attenuate regulatory signals (71, 72).

SP1 is a ubiquitous transcription factor that regulates genes involved in cell proliferation, apoptosis, and immune response (87). STAT3 is an important transcription factor in cytokine signaling and in regulating both innate and adaptive immune responses (87–89). In the context of tuberculosis, STAT3 has been implicated in macrophage activation and in the response to interferon-gamma (IFN-γ), a cytokine essential for controlling *Mtb* (87).

Previous studies have shown that polymorphisms in the STAT3 gene have been associated with susceptibility in human populations, and their dysfunction compromises innate immunity against mycobacteria (87). In patients with TB-T2DM comorbidity, hyperglycemia induces aberrant STAT3 phosphorylation, contributing to immune dysfunction (89).

Experimental studies indicate that phosphorylated STAT3 suppresses specific miRNAs (such as miR-19b and miR-1281), worsening lung damage in mouse models of DM2-associated TB (89).

MYC is a proto-oncogene that regulates genes involved in cell cycle, metabolism, and apoptosis. In tuberculosis, MYC expression has been linked to dysregulation of macrophage antimicrobial activity in controlling *Mtb infection* (90).

BCL2 is an anti-apoptotic regulator that protects cells from apoptosis, and its dysregulation can affect infected cell survival and immune responses (66, 78).

PTEN is a tumor suppressor that antagonizes the PI3K/AKT pathway, modulating insulin signaling and glucose metabolism, processes central of T2DM pathogenesis.

The identification of EGFR (Epidermal Growth Factor Receptor) as a hub gene suggests a connection between growth factor signaling and response to infection and metabolism. CDK4 (Cyclin-Dependent Kinase 4) regulates cell cycle progression, while SMAD4 mediates signaling by TGF-β, a cytokine with complex roles in immunity and fibrosis. NAA50 and CFL2 are less characterized in the context of TB-T2DM, but their identification as central nodes warrants further investigation.

### Target gene protein-protein interaction network

To further characterize the functional interactions of the target genes of overexpressed miRNAs, a protein-protein interaction (PPI) was constructed using the STRING database. The centrality analysis of this PPI network identified 10 genes with high centrality: *STAT3, MYC, BCL2, AKT1, CTNNB1, JUN, IL-6, TP53, TNF*, and *HIF1A* (**Figure 6B**).

These hub genes represent regulators involved in both tuberculosis immunity and the pathophysiology of type 2 diabetes mellitus. AKT1 is a central component of the PI3K/AKT signaling pathway, essential for insulin signaling, glucose and lipid metabolism, and cell survival.

The AKT pathway regulates GLUT4 and GLUT1-mediated glucose transport, glycogen synthesis, hepatic gluconeogenesis, and lipogenesis, processes altered in T2DM.

In addition, AKT1 also regulates pancreatic β cell function, regulating insulin secretion and the adaptive stress response of the endoplasmic reticulum.

In the context of tuberculosis, AKT signaling in macrophages modulates phagocytosis, production of reactive oxygen and nitrogen species, and response to proinflammatory cytokines (66, 78).

HIF1A is a transcription factor that regulates the cellular response to hypoxia, a condition characteristic of the granuloma microenvironment in the tuberculous (75–77, 91, 92). HIF-1α coordinates metabolic adaptation toward aerobic glycolysis, increases glucose transporter expression (GLUT1), and regulates proinflammatory cytokine production (93–96).

In macrophages infected with *Mtb*, HIF-1α is essential for IFN-γ-induced activation and for the control of intracellular bacterial replication (93, 94). Stabilization of HIF-1α under conditions of granulomatous hypoxia promotes antimicrobial mechanisms, including autophagy and antimicrobial peptide production (83, 93, 94). However, in the context of T2DM, hyperglycemia and carbonyl stress (e.g., methylglyoxal, MGO) impair HIF-1α-regulated responses, compromising the control of *Mtb* in macrophages (83).

Experimental studies indicate that desferrioxamine (DFO), a stabilizer of HIF-1α, restores mycobacterial control under high-glucose conditions, suggesting HIF-1α as a potential therapeutic target to enhance the immune response in patients with T2DM-TB (83). In addition, HIF-1α regulates the expression of matrix metalloproteinases (MMPs) in hypoxic macrophages infected with *Mtb*, contributing to tissue damage and the formation of lung cavities characteristic of advanced tuberculosis (97).

TNF and IL-6 are key proinflammatory cytokines in tuberculosis immunity and T2DM pathogenesis (66, 76, 78–80, 92). TNF is essential for the formation and maintenance of granulomas, the recruitment of immune cells, and the activation of macrophages (76, 92).

However, excessive or dysregulated TNF production contributes to immunopathology, tissue necrosis, and cachexia (76, 92). In diabetic TB patients, TNF levels are frequently elevated, contributing to a state of chronic inflammation that exacerbates both insulin resistance and lung tissue damage (66, 78). IL-6 regulates the acute-phase response, B and T cell activation, and Th17 cell differentiation, and elevated IL-6 levels are associated with insulin resistance and diabetic complications (66, 92).

TP53 is a tumor suppressor that regulates the cellular stress response, apoptosis, and senescence. JUN is a component of the transcription factor AP-1, involved in proliferation, differentiation, and stress response. CTNNB1 (β-catenin) is a mediator of Wnt signaling, with roles in development, tissue homeostasis, and metabolism.

The convergence of these hub genes into a densely interconnected network suggests that the overexpressed miRNAs shared between TB and T2DM function as pleiotropic regulators, simultaneously modulating key cellular processes, such as insulin signaling (AKT1, PTEN), hypoxia response (HIF1A), inflammation (TNF, IL-6), apoptosis (BCL2, TP53), and metabolic adaptation. Convergence of these hub genes into a densely interconnected network suggests that overexpressed miRNAs shared between TB and T2DM function as pleiotropic regulators, simultaneously modulating key cellular processes such as insulin signaling (AKT1, PTEN), hypoxia response (HIF1A), inflammation (TNF, IL6), apoptosis (BCL2, TP53), and metabolic adaptation. Network architecture offers a framework for understanding how miRNA dysregulation may contribute to TB–T2DM comorbidity, connecting metabolic dysfunction with impaired immune responses.

### Integrated biological interpretation

Taken together, the bioinformatic results suggest that the overexpressed miRNAs shared between TB and T2DM repress genes essential for the innate and adaptive immune response against *Mtb*. Suppression of processes such as leukocyte adhesion, migration, and differentiation reduces the host’s capacity to recruit effector cells from granulomas and eliminate intracellular bacteria (65–67, 78, 79) (**Table 4**). At the same time, modulation of central metabolic pathways (glucose metabolism, insulin signaling, response to hypoxia) sustains a state of metabolic-immunological dysfunction that promotes both T2DM progression and TB reactivation and dissemination (66, 79, 80, 83).

**Table 4:** Top immunological processes and the hub genes related to tuberculosis.

Biological Process	P adjusted	Genes Hub
Myeloid leukocyte differentiation	4.61E-07	MYC, TNF, CTNNB1, JUN
Response to molecule of bacterial origin	1.69E-05	CDK4, TNF, AKT1, IL6
Positive regulation of leukocyte migration	0.00029956	TNF, IL6
Leukocyte apoptotic process	0.00052312	BCL2, MYC, HIF1A, AKT1, TP53, IL6
Regulation of leukocyte differentiation	0.00073687	MYC, TNF, CTNNB1
Positive regulation of leukocyte chemotaxis	0.00095156	IL6
T cell differentiation involved in immune response	0.00095156	STAT3, IL6
Regulation of myeloid leukocyte differentiation	0.00095156	MYC, TNF, CTNNB1
Leukocyte cell adhesion	0.00095156	TNF, AKT1, IL6

In addition, inhibition of apoptosis-related genes contributes to infection spread and poorer prognosis in patients with active TB (98).

Finally, these findings highlight the importance of systems biology approaches to understand complex diseases with multiple comorbidities, where the interaction among miRNA, mRNA, proteins, and metabolic pathways generates emergent properties not predictable from the study of individual components (17, 65–69).

**Figure 7:**
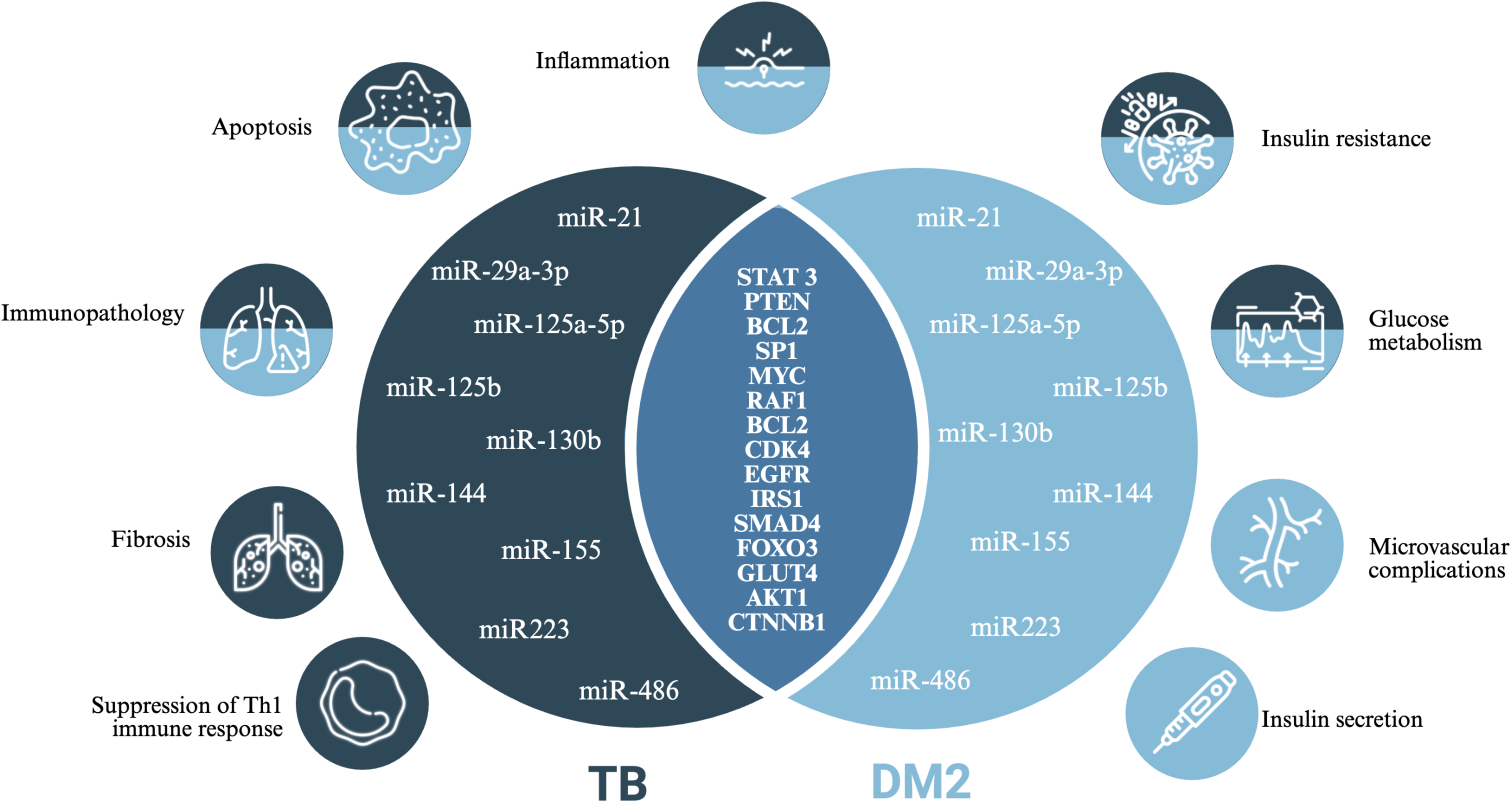
Showing the shared dysregulated microRNAs between TB and T2DM, along with their experimentally validated target genes and associated biological processes. The representation demonstrates the convergence of immunological and metabolic pathways regulated by hsa-miR-21, hsa-miR-29a-3p, hsa-miR-125a-5p, hsa-miR-125b, hsa-miR-130b, hsa-miR-144, hsa-miR-155, hsa-miR-223, and hsa-miR-486. The main target genes include STAT3, PTEN, BCL2, SP1, MYC, RAF1, CDK4, EGFR, IRS1, SMAD4, FOXO3, GLUT4, AKT1, and CTNNB1, involved in immune regulation processes, insulin signaling, glucose metabolism, apoptosis, fibrosis, and microvascular complications.

**Figure 7** synthesizes the convergent molecular mechanisms identified in this study, showing dysregulated miRNAs shared between TB and T2DM along with their experimentally validated target genes and associated biological processes.

## Discussion

The present systematic review identified a set of miRNAs whose expression is jointly dysregulated in patients with pulmonary TB and T2DM and characterized the molecular networks through which these miRNAs modulate immunological and metabolic processes critical for the pathophysiology of both diseases. To our knowledge, the present study is the first to carry out a systematic review, analyzing the miRNAs that are involved in the TB-T2DM comorbidity. Currently, the shared biological mechanisms underlying both diseases are not fully elucidated. Therefore, there is a need to find prognostic, diagnostic, or follow-up biomarkers or molecules that can represent potential molecular targets.

The main findings of this study reveal that shared overexpressed miRNAs negatively regulate genes involved in leukocyte adhesion, migration, differentiation, and cell death, as well as in the specific immune response against TB. The network analysis identified high-centrality hub genes, including STAT3, AKT1, HIF1A, TNF, IL-6, BCL2, MYC, and PTEN, that constitute critical nodes of integration between metabolism and immunity. These genes represent potential therapeutic targets for host-directed therapies in patients with TB-DM2 comorbidity (65, 66, 75, 83, 87, 89, 93). For example, restoring HIF-1α function using pharmacological stabilizers, modulating AKT signaling to improve both insulin sensitivity and macrophage function, and balanced regulation of proinflammatory cytokines such as TNF and IL-6 are promising strategies that deserve exploration in future preclinical and clinical studies (66, 80, 83). Our results provide a robust conceptual framework for understanding the molecular mechanisms that link the metabolic dysfunction characteristic of T2DM with immunosuppression that favors TB reactivation and progression and suggest multiple translational applications of clinical and public health relevance.

Circulating miRNAs have emerged in the last decade as promising molecular biomarkers for the diagnosis of infectious and metabolic diseases due to their unique biological properties: high stability in body fluids (serum, plasma, urine, sputum), resistance to degradation by RNases, tissue- and disease-specific expression profiles, and accessibility through non-invasive detection techniques (99–103). In the context of TB, multiple studies have shown that specific miRNAs such as miR-29a, miR-155, miR-425-5p, let-7d-5p, and miR-140-5p can discriminate between tuberculosis infection, active TB, and healthy controls with sensitivities ranging from 78% to 94% and specificities ranging from 76% to 92% (26, 43, 101, 104–107). Similarly, in T2DM, circulating miRNAs such as miR-126, miR-122, miR-146a, miR-192, and miR-194 have shown diagnostic and prognostic utility, predicting progression from prediabetes to established diabetes and stratifying the risk of microvascular and macrovascular complications (108–114). Our study highlights hsa-miR-155-5p and hsa-miR-29a-3p, which are overexpressed in TB and T2DM and appear to be central regulators of extracellular matrix adhesion and remodeling. Previous reports indicate that hsa-miR-29a-3p is linked to inhibition of cell adhesion and migration. Specifically, this miRNA decreases the expression of genes such as VCAM-1, ICAM-1, and E-cadherin in endothelial cells, which affects the adhesion of leukocytes (74). With respect to hsa-miR-155-5p, it has been reported that the overexpression of this miRNA in bone marrow-derived neutrophils favors NETosis, increasing the damage caused by inflammation exacerbated by this type of cell death (115). NETosis, and its molecular markers, is associated with granuloma destruction and caseous center formation in patients with active TB (116). The findings reported in the present study suggest that hsa-miR-155-5p and hsa-miR-29a-3p could be potential molecular targets in the TB-T2DM comorbidity, as they regulate key cellular processes that are involved in TB immunopathology. However, further studies are required to better understand the involvement of these miRNAs in TB and T2DM.

The identification of deregulated miRNAs shared between TB and T2DM in the present study opens innovative perspectives for the development of integrated diagnostic biomarkers specific to TB-T2DM comorbidity. These biomarkers could facilitate bidirectional screening, identifying TB patients who are at high risk of developing undiagnosed T2DM or prediabetes and detecting diabetic patients at high risk of ITL reactivation or progression to active TB (117–121). This early detection strategy is particularly relevant in contexts with a high burden of both diseases, where diagnostic resources are limited and underdiagnosis rates are high (119–121).

From a technical perspective, the development of diagnostic panels based on multiple miRNAs has demonstrated superior performance to single miRNAs in studies of TB and other diseases (43, 122, 123). Similarly, a panel combining let-7d-5p and miR-140-5p distinguished ITL from active TB with AUC of 0.93 and 100% sensitivity (43). These results demonstrate that the rational design of multimiRNA panels, integrating differential expression information, network analysis, and functional validation, can significantly optimize diagnostic performance (43, 101, 107).

In the context of TB-T2DM comorbidity, we propose that the shared miRNAs identified in this study, particularly those that regulate hub genes such as STAT3, AKT1, HIF1A, TNF, and IL-6, could constitute priority candidates for the development of molecular signatures of high diagnostic and prognostic value. The measurement of these miRNAs in serum or plasma samples using ultra-sensitive techniques such as Single Molecule Arrays (Simoa) or real-time quantitative PCR (RT-QPCR) would allow patients to be stratified according to risk profiles, to identify subgroups that would benefit from preventive interventions (LTI treatment in diabetics, intensive glycemic control in TB patients), and to monitor the response to antituberculosis and antidiabetic treatment in an integrated manner (101, 105, 107, 124).

A critical aspect for the clinical translation of these biomarkers is methodological standardization and validation in prospective multicenter cohorts. Previous studies have identified important challenges related to the normalization of circulating miRNA levels, the selection of appropriate endogenous controls (such as miR-93 in TB), inter-assay variability, and the need to consider demographic and clinical confounders (age, sex, body mass index, concomitant drug treatments) that may affect miRNA profiles (102, 125–127). Overcoming these technical challenges through standardized protocols and rigorous quality controls is essential to ensure the reproducibility and reliability of miRNA-based biomarkers (100, 102, 125).

In addition, the integration of miRNA profiling with other molecular biomarkers such as inflammatory cytokines (IFN-γ, TNF, IL-6), acute phase markers (C-reactive protein, ferritin), and biomarkers of metabolic function (glycosylated hemoglobin HbA1c, C-peptide, insulin resistance indices) could generate high-throughput, multiparametric diagnostic algorithms that capture the multidimensional complexity of TB-T2DM comorbidity (17, 66, 120). Such algorithms, implemented in point-of-care molecular diagnostic platforms, would have a transformative impact in resource-limited contexts, where access to specialized laboratories is restricted and the need for fast, accurate, and inexpensive diagnostics is critical (102, 120, 128).

**Figure 8:**
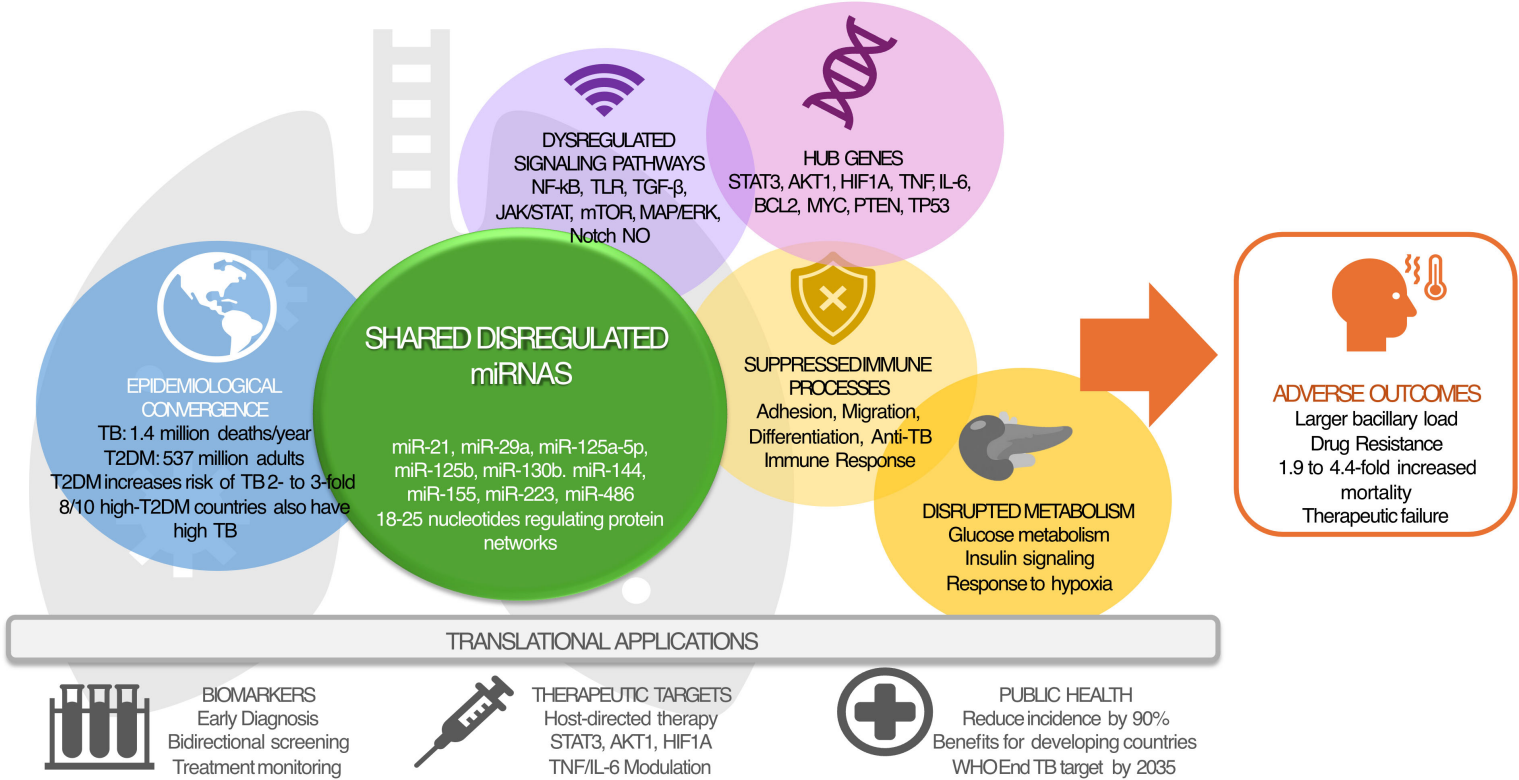
The interaction between TB and T2DM is explained not only by epidemiological convergence but also by shared molecular mechanisms that compromise the immune response and cell metabolism. miRNA dysregulation is a central axis of this comorbidity. The main miRNAs identified in this study of TB and T2DM patients include miR-21, miR-29a, miR-125a-5p, miR-125b, miR-130b, miR-144, miR-155, miR-223, and miR-486, all of them with critical functions in the modulation of signaling pathways such as NF-κB, JAK/STAT, mTOR, and TGF-β. These miRNAs act as post-transcriptional regulators of key genes (STAT3, AKT1, HIF1A, TNF, IL-6, TP53, PTEN), affecting processes of cell differentiation, adhesion, and migration, as well as insulin signaling and response to hypoxia. Their altered profile in TB-DM2 comorbidity suggests a dual role: on the one hand, they contribute to immunosuppression and the greater susceptibility of diabetic patients to developing active TB; on the other, they offer translational potential as genetic biomarkers for early diagnosis, risk stratification, and therapeutic monitoring. The integration of the figure allows us to encompass how miRNA dysregulation translates into alterations of immunometabolic networks and adverse clinical outcomes. This combined approach reinforces the need to implement bidirectional screening strategies and to explore host-directed therapies that modulate these molecular nodes.

**Figure 8** visually summarizes the main findings of this systematic review and bioinformatics analysis, integrating into a single diagram the epidemiological, molecular, and translational elements that link TB and T2DM. The top portion highlights the epidemiological convergence between the two diseases, underscoring the high prevalence in low- and middle-income countries and the increased risk of TB in patients with T2DM. The center illustrates the shared microRNAs (miR-21, miR-29a, miR-144, miR-155, miR-223, among others), along with the immunometabolic signaling pathways they regulate (NF-κB, TLR, JAK/STAT, mTOR), and the hub genes identified through network analysis (STAT3, AKT1, HIF1A, TNF, IL-6, TP53), reflecting the common molecular architecture between the two pathologies. At the extremes, the altered immunological and metabolic processes are shown, such as cell adhesion, migration, insulin signaling, and the response to hypoxia, which explain the adverse clinical outcomes observed in TB-T2DM comorbidity. Finally, at the bottom, the translational applications of these findings are summarized: from the development of biomarkers for early diagnosis and therapeutic monitoring to the design of host-directed therapies and public health strategies aimed at reducing the global burden of TB. This figure allows for an integrated visualization of how shared miRNAs act as central nodes in the immunometabolic interaction between TB and T2DM, offering new opportunities for personalized medicine and preventive intervention.

The absence of a weighting system constitutes a methodological limitation of this work. Our approach was aimed at identifying recurrent nodes of interaction between miRNAs and target genes, with the purpose of building a conceptual map of the shared mechanisms in TB-DM2 comorbidity. However, we recognize that incorporating quantitative metrics such as effect size and sample size would strengthen the validity of the conclusions and provide a more robust synthesis. In this sense, future studies should integrate these metrics into their analyses to more accurately assess the biological and clinical relevance of the identified miRNAs.

This paper has some limitations that should be considered when interpreting the findings. First, the methodological heterogeneity of the included studies in terms of sample size, population characteristics, and miRNA detection techniques may introduce bias and limit the direct comparability of the results. Second, although the PRISMA 2020 guidelines were followed, a standardized risk of bias assessment tool was not applied, which restricts critical assessment of the quality of the primary evidence. Likewise, the analysis was limited to studies conducted in adult humans. Finally, the results are based on bioinformatic analyses without their own clinical validation, so the proposed diagnostic or therapeutic applications should be considered preliminary and require confirmation in prospective and functional studies.

## Conclusion

In the present study, altered miRNAs in TB and T2DM were analyzed through a bioinformatic analysis of a systematic review of the literature published to date. The miRNAs that are shared and overexpressed in both pathologies establish a complex network with their respective target genes, which regulates biological and immunological processes that are important in the response to infection with the mycobacterium. The findings of this research provide new knowledge in the understanding of the complex relationship between the two diseases, clarifying the molecular mechanisms involved in the immunopathology of this binomial.

The translation of our findings into clinical applications could have a significant impact on the reduction of morbidity and mortality associated with this comorbidity, since the identified miRNAs, as well as their molecular targets, could be used as molecular targets and as diagnostic or predictive biomarkers. These findings will be of great interest to contribute to the sustainability of health systems and the achievement of the health-related sustainable development goals.

## Data Availability

The original contributions presented in the study are included in the article/supplementary material. Further inquiries can be directed to the corresponding author.
